# Impact of Total and Positive Lymph Node Count on Recurrence in Young Women with Node-Positive Breast Cancer Undergoing Neoadjuvant Chemotherapy

**DOI:** 10.1245/s10434-026-20154-w

**Published:** 2026-07-08

**Authors:** Lucy Zhang, Shama Birla, Sophia X. Tang, Olivia W. Galloway, Sydney Kinkade, Ariana Naaseh, Omolade O. Sogade, Julie A. Margenthaler

**Affiliations:** Section of Surgical Oncology, Department of Surgery, Washington University School of Medicine in St. Louis, St. Louis, MO USA

**Keywords:** Breast cancer, Neoadjuvant chemotherapy, Young women, Lymph node ratio

## Abstract

**Background:**

Axillary management after neoadjuvant chemotherapy (NAC) in node-positive breast cancer remains controversial. This study assessed the association between total lymph node count, in the context of metastatic lymph node count and lymph node ratio (LNR), and recurrence after NAC in a cohort of young women with node-positive breast cancer.

**Methods:**

Retrospective data were analyzed from 274 women aged ≤ 45 years diagnosed with clinical T1–3, N1–3, M0 invasive breast cancer between January 2010 and December 2023 who received NAC before undergoing surgery at our institution. Primary outcomes were recurrence and overall survival. Total lymph node counts across three LNR tiers were compared between women with at least one metastatic node out of at least three retrieved nodes who did and did not develop recurrence.

**Results:**

Patients who developed a recurrence presented with more advanced clinical T (*p* < 0.001) and N (p = 0.01) staging and had greater residual burden of disease in both the breast (*p *< 0.001) and the lymph nodes (*p* < 0.001) than did patients with no recurrence. There was no significant correlation between total number of lymph nodes excised and recurrence in women with at least one metastatic node out of at least three examined nodes in any of the low (*p* = 0.39), intermediate (*p* = 0.47), or high (*p* = 0.67) LNR tiers.

**Conclusion:**

Tumor biology, chemoresistance, and residual tumor burden after NAC may influence oncologic outcomes more than the number of nodes removed or the extent of nodal surgery. Prospective, large-scale, long-term studies are needed to rigorously address this question, particularly in this young, high-risk population.

**Supplementary Information:**

The online version contains supplementary material available at https://doi.org/10.1245/s10434-026-20154-w.

Neoadjuvant chemotherapy (NAC) is used in breast cancer to downstage bulky disease before surgery and allows clinicians to evaluate both tumor chemoresistance and patient prognosis. This is especially relevant in human epidermal growth factor receptor 2 (HER2)-positive and triple-negative breast cancer (TNBC). For instance, over 60% of patients with TNBC experience a pathologic complete response (pCR) when treated with NAC in combination with the immunotherapy pembrolizumab, and 3-year recurrence-free survival approaches 99% among patients with HER2+ disease who experience a pathologic complete response after NAC.^1,2^

Optimal approaches to axillary management after NAC in patients with node-positive disease are widely debated. National Comprehensive Cancer Network guidelines support sentinel lymph node biopsy (SLNB) in patients with clinically positive axillary nodes that clear after NAC, and false-negative rates are improved to <10% with the use of dual-tracer mapping and retrieval of at least three nodes, including any biopsy-proven, clipped node.^3–7^ Current guidelines recommend axillary lymph node dissection (ALND) as a treatment option in patients with persistent nodal disease after NAC.^3^ However, this paradigm is currently being challenged, and emerging evidence indicates there are no significant differences in locoregional or distant recurrence following SLNB as compared with ALND among most ypN+ and ypN1mi patients.^8,9^

Young patients with breast cancer aged <45 years represent a unique clinical population that faces specific challenges related to genetic counseling, fertility preservation, psychosocial factors, and survivorship and exhibit distinct differences in tumor biology. Young women are more likely to present with higher grade, later stage, and more aggressive breast cancers with poorer prognostic features, including TNBC or HER2+ subtypes.^10–12^

Current nodal prognostication staging, such as pathologic N or ypN staging after neoadjuvant therapy, does not take into consideration the total number of lymph nodes removed. Lymph node ratio (LNR) is the proportion of pathologically positive lymph nodes to total nodes retrieved. LNR has been evaluated in many oncologic contexts, including colorectal, lung, pancreatic, and breast, and greater LNR is associated with a worse prognosis.^13–17^ To our knowledge, no study to date has investigated the relationship between lymph node counts across LNRs and oncologic outcomes: is there a difference in recurrence between patients with, for instance, three of three positive nodes retrieved versus 23 of 23 positive nodes? We assessed the association between total lymph node count, in the context of metastatic lymph node count and LNR, and recurrence and survival outcomes after NAC in a cohort of young women with node-positive breast cancer treated at our institution.

## Methods

### Study Population

The study protocol (#202306033) was deemed exempt by the Washington University institutional review board. A database of 757 women aged ≤45 years referred to breast surgical oncology at BJC HealthCare/WashU Medicine who received NAC and, when appropriate, systemic targeted therapy was retrospectively reviewed. Patients with clinical T1–3, N1–3, M0, unilateral, invasive breast cancer and who underwent breast biopsy between January 2010 and December 2023 were identified. Clinical nodal staging was determined based on either positive percutaneous biopsy results or the presence of suspicious lymph nodes on physical examination or imaging (ultrasound, magnetic resonance imaging, or computed tomography when performed).

### Variables

Data on patient age, race, and ethnicity were extracted, as were data on tumor receptor subtype (TNBC, HER2+, or hormone receptor [HR]+/HER2-) and clinical and pathologic T and N staging. Data on the type of breast surgery (mastectomy vs. lumpectomy) and axillary surgery (SLNB vs. ALND) that patients underwent, as documented in the operative note, and whether they received adjuvant chemo/immunotherapy, radiation therapy, and endocrine therapy, were also recorded. Histopathologic findings, including total examined lymph node count and positive lymph node count, were collected. A lymph node with any macrometastasis or micrometastasis was counted as a positive node. Nodes with isolated tumor cells were documented separately. Median follow-up in months was recorded.

### Statistical Analysis

Statistical analysis was performed using the X^2^ and Fisher’s exact tests for categorical variables; Mann–Whitney U and exact Wilcoxon–Mann–Whitney tests for continuous variables; and Student’s unpaired t-test. Exact tests were used for comparisons with n ≤ 5. Data were analyzed using IBM SPSS Statistics (version 31.0.1.0). Statistical significance was set at *p* < 0.05.

### Outcomes

The primary outcomes were recurrence, whether locoregional, contralateral, or distant, and overall survival. Median time to recurrence and survival time in months was analyzed. Additionally, we examined LNR among patients who had one or more metastatic node out of three or more retrieved sentinel nodes; in other words, patients with zero metastatic nodes (LNR = 0) were excluded from the LNR analysis, as were any patients with only one or two total nodes retrieved. In this subgroup, total examined lymph node counts across three LNR tiers (Low: 0.01–0.34; intermediate: 0.35–0.67; high: 0.68–1.0) were compared between those who did and did not develop recurrence.

## Results

Of the 274 patients who met the inclusion criteria, 196 (71.5%) did not develop a recurrence. There were no significant demographic differences between patients who developed a recurrence and those who did not (Table 1). Overall, the median age was 37 years (interquartile range [IQR] 33–41), with 60.9% (167 patients) aged 36–45 years at the time of diagnosis. The majority of patients were white (73.4%, n = 201) and non-Hispanic (90.5%, n = 248).

**Table 1:** Patient demographics

Characteristics	Overall n=274	Recurrence n=78 (28.5%)	No recurrence n=196 (71.5%)	*p*-Value
Age, years	37 (33–41)	37 (31–40)	38 (34–41)	0.11
*Age group, years*				0.67
≤35	107 (39.1)	32 (41.0)	75 (38.3)	
36–45	167 (60.9)	46 (59.0)	121 (61.7)	
*Race*				0.53
Asian	4 (1.5)	1 (1.3)	3 (1.5)	
Black	67 (24.5)	23 (29.5)	44 (22.4)	
White	201 (73.4)	54 (69.2)	147 (75.0)	
Declined/other	2 (0.7)	0 (0.0)	2 (1.0)	
*Ethnicity*				0.88
Hispanic	8 (2.9)	2 (2.6)	6 (3.1)	
Non-Hispanic	248 (90.5)	70 (89.7)	178 (90.8)	
Not specified	18 (6.6)	6 (7.7)	12 (6.1)	

Data are presented as n (%) or median (interquartile range) unless otherwise indicated

Tumor characteristics, including receptor status, clinical T and N stage, pathologic T and N stage, and type of breast and axillary surgery performed, are summarized in Table 2. There was no significant difference in tumor receptor status between the groups that did and did not suffer a recurrence (*p* = 0.16). Most patients who developed a recurrence had TNBC (34.6%, n = 27) or HR+/HER2- (46.2%, n = 36) cancers.

**Table 2:** Tumor characteristics and details of surgical treatment

Characteristics	Overall n=274	Recurrence n=78 (28.5%)	No recurrence n=196 (71.5%)	*p*-Value
*Receptor status*				0.16
TNBC	86 (31.4)	27 (34.6)	59 (30.1)	
HER2+	75 (27.4)	15 (19.2)	60 (30.6)	
HR+/HER2-	113 (41.2)	36 (46.2)	77 (39.3)	
*Clinical T stage*				**<0.001**
T1	23 (8.4)	5 (6.4)	18 (9.2)	
T2	148 (54.0)	27 (34.6)	121 (61.7)	
T3	103 (37.6)	46 (59.0)	57 (29.1)	
*Clinical N stage*				**0.01**
N1	221 (80.7)	55 (70.5)	166 (84.7)	
N2	18 (6.6)	6 (7.7)	12 (6.1)	
N3	35 (12.8)	17 (21.8)	18 (9.2)	
*ypT stage*				**<0.001**
ypT0	89 (32.5)	10 (12.8)	79 (40.3)	
ypTis	13 (4.7)	4 (5.1)	9 (4.6)	
ypT1	105 (38.3)	31 (39.7)	74 (37.8)	
ypT2	47 (17.2)	20 (25.6)	27 (13.8)	
ypT3	17 (6.2)	10 (12.8)	7 (3.6)	
ypT4	3 (1.1)	3 (3.8)	0 (0.0)	
*ypN stage*				**<0.001**
ypN0	140 (51.1)	19 (24.4)	121 (61.7)	
ypN0(i+)	5 (1.8)	1 (1.3)	4 (2.0)	
ypN1	92 (33.6)	36 (46.2)	56 (28.6)	
ypN2	28 (10.2)	14 (17.9)	14 (7.1)	
ypN3	9 (3.3)	8 (10.3)	1 (0.5)	
*Breast surgery*				0.67
Lumpectomy	68 (24.8)	18 (23.1)	50 (25.5)	
Mastectomy	206 (75.2)	60 (76.9)	146 (74.5)	
*Axillary surgery*				0.07
SLNB	154 (56.2)	37 (47.4)	117 (59.7)	
ALND	120 (43.8)	41 (52.6)	79 (40.3)	
*Examined lymph node count*				
Mean ± SD	7.9 ± 6.2	8.8 ± 6.7	7.6 ± 5.9	0.14
Median (IQR)	6 (3–12)	7 (4–13)	5 (3–11)	0.15
1	14 (5.1)	3 (3.8)	11 (5.6)	
2	29 (10.6)	7 (9.0)	22 (11.2)	
3	42 (15.3)	9 (11.5)	33 (16.8)	
4	27 (9.9)	10 (12.8)	17 (8.7)	
5–9	70 (25.5)	18 (23.1)	52 (26.5)	
10–15	58 (21.2)	21 (26.9)	37 (18.9)	
≥16	34 (12.4)	10 (12.8)	24 (12.2)	
Positive lymph nodes	0 (0–2)	2 (1–4)	0 (0–1)	**<0.001**

Bold values are statistically significant (*p* < 0.05)

Data are presented as n (%) or median (interquartile range) unless otherwise indicated

ALND, axillary lymph node dissection; HER2, human epidermal growth factor receptor 2; IQR = interquartile range; HR, hormone receptor; SD, standard deviation; SLNB, sentinel lymph node biopsy; TNBC, triple-negative breast cancer

Clinical T3 disease (58.9%, n = 46) was dominant among patients who developed a recurrence, followed by T2 (34.6%, n = 27) disease. In contrast, clinical T2 disease was most common (61.7%, n = 121) among patients who did not develop a recurrence, followed by T3 disease (29.1%, n = 57). This difference in the distribution of clinical T staging between patients who did and did not suffer a recurrence was significant (p < 0.001). Most patients across both groups had clinical N1 disease (80.7%, n = 221), though clinical N3 disease was more common among those who developed a recurrence than among those who did not (21.8% vs 9.2%). There was also a significant difference in the distribution of clinical N staging between the groups that did and did not recur (p = 0.01). After undergoing NAC, patients who developed a recurrence had a greater proportion of ypT2-3 (38.5% vs. 17.3%) and ypN1-3 (74.4% vs. 36.2%) disease than those who did not develop a recurrence. Notably, 61.7% (n = 121) of patients who did not develop a recurrence achieved ypN0 status following NAC, as compared with only 24.4% (n = 19) of women who did develop a recurrence; the distribution of ypN staging was significant between the recurrence and no-recurrence groups (*p* < 0.001). Overall, 75.2% (n = 206) of patients underwent mastectomy, and 56.2% (n = 154) underwent SLNB. There were no statistically significant differences in the type of breast or axillary surgical treatment between patients who developed a recurrence and those who did not (*p* = 0.67 and *p* = 0.07, respectively).

An overall average of 7.9 (standard deviation [SD] 6.2) lymph nodes was removed during surgery, whether SLNB or ALND. A median of two metastatic nodes (IQR 1–4) were excised from those who developed a recurrence, as compared with 0 (IQR 0–1) from those who did not (*p* < 0.001).

Adjuvant therapy and recurrence and survival outcomes are detailed in Table 3. Overall, approximately half of the patients received adjuvant chemo/immunotherapy (49.6%, n = 136), and most patients received adjuvant radiation therapy (87.6%, n = 240). There were no significant differences in rates of adjuvant chemo/immunotherapy (*p* = 0.57) or radiation therapy (*p* = 0.18) between patients who did and did not develop a recurrence. Rates of adjuvant endocrine therapy were significantly different between patients who did and did not develop a recurrence (*p* = 0.04); 44.9% (n = 35) of those who developed a recurrence and 58.2% (n = 114) of those who did not received adjuvant endocrine therapy .

**Table 3:** Adjuvant therapies and recurrence and survival outcomes

Therapy and outcomes	Overall n=274	Recurrence n=78 (28.5%)	No recurrence n=196 (71.5%)	*p*-Value
*Adjuvant chemo/immunotherapy*^*a*^				0.57
Yes	136 (49.6)	41 (52.6)	95 (48.5)	
No	137 (50.0)	37 (47.4)	100 (51.0)	
*Adjuvant radiation therapy*				0.18
Yes	240 (87.6)	65 (83.3)	175 (89.3)	
No	34 (12.4)	13 (16.7)	21 (10.7)	
*Adjuvant endocrine therapy*^*b*^				**0.04**
Yes	149 (54.4)	35 (44.9)	114 (58.2)	
No	123 (44.9)	43 (55.1)	80 (40.8)	
Follow-up, months	60 (31–99)	43 (25.25–82.75)	62.5 (35–103)	**0.01**
Months to recurrence	NA	21 (14.25–42.75)	NA	
*Type of recurrence*				
Locoregional recurrence	NA	18 (23.1)	NA	
Contralateral breast	NA	0 (0.0)	NA	
Distant metastasis	NA	60 (76.9)	NA	
*Death*				**<0.001**
Yes	57 (20.8)	53 (67.9)	4 (2.0)	
No	217 (79.2)	25 (32.1)	192 (98.0)	
Survival, months^c^	40 (21.75–62.75)	40 (21–64.25)	35.5 (24.75–50.25)	0.92

Bold values are statistically significant (*p* < 0.05)

Data are presented as n (%) or median (interquartile range) unless otherwise indicated

^a^No data available for one patient

^b^No data available for two patients

^c^No data available for three patients

Overall median follow-up was 60 months (IQR 31–99). Median time to recurrence was 21 months (IQR 14.25–42.75). In total, 76.9% (n = 60) of recurrences were distant metastases. There was a significant difference in survival (*p* < 0.001) between both groups; 67.9% (n = 53) of patients who developed a recurrence died at a median follow-up of 43 months (IQR 25.25–82.75), whereas only 2% (n = 4) of patients who did not develop a recurrence died at a median follow-up of 62.5 months (IQR 35–103). Among patients who died during the follow-up period, median survival duration was not significantly different between patients who did and did not develop a recurrence (40 vs. 35.5 months, *p* = 0.92).

LNR was defined as the ratio of positive lymph nodes to total lymph nodes retrieved and was calculated for patients with at least three lymph nodes examined and at least one positive lymph node (n = 120). There were 140 patients with zero metastatic nodes, and 43 patients with only one or two nodes removed, of which 14 patients had one of one, one of two, or two of two positive nodes. The mean LNR overall was 0.39 (SD: ± 0.27), Table 4. Mean LNR was significantly different (*p* = 0.01) between patients who developed a recurrence (0.49 ± 0.32) and those who did not (0.37 ± 0.24). The median LNR was 0.33 (IQR 0.20–0.53) overall, 0.43 (IQR 0.23–0.76) among patients who developed a recurrence, and 0.33 (IQR 0.21–0.50) among patients who did not develop a recurrence (*p* = 0.05).

**Table 4:** Lymph node ratios (LNRs)

	Overall n=120	Recurrence n=52 (43.3%)	No recurrence n=68 (56.7%)	*p*-Value
*LNR*				
Mean	0.39 ± 0.27	0.49 ± 0.32	0.37 ± 0.24	**0.01**
Median	0.33 (0.20–0.53)	0.43 (0.23–0.76)	0.33 (0.21–0.50)	0.05
*LNR 0.01–0.34*				0.39
3 lymph nodes examined	14	2 (8.0)	12 (24.5)	
4	6	3 (12.0)	3 (6.1)	
5–9	20	6 (24.0)	14 (28.6)	
10–15	22	9 (36.0)	13 (26.5)	
≥16	12	5 (20.0)	7 (14.3)	
Total	74	25 (33.8)	49 (66.2)	
*LNR 0.35–0.67*				0.47
3 lymph nodes examined	3	0 (0.0)	3 (21.4)	
4	3	1 (7.7)	2 (14.3)	
5–9	7	4 (30.8)	3 (21.4)	
10–15	11	7 (53.8)	4 (28.6)	
≥16	3	1 (7.7)	2 (14.3)	
Total	27	13 (48.1)	14 (51.9)	
*0.68–1.0*				0.67
3 lymph nodes examined	3	2 (14.3)	1 (20.0)	
4	4	2 (14.3)	2 (40.0)	
5–9	5	4 (28.6)	1 (20.0)	
10–15	3	2 (14.3)	1 (20.0)	
≥16	4	4 (28.6)	0 (0.0)	
Total	19	14 (73.7)	5 (26.3)	

Bold values are statistically significant (*p* < 0.05)

Data are presented as mean ± standard deviation, median (interquartile range), or n (%) unless otherwise indicated

These patients were stratified to low LNR (0.01–0.34, n = 74), intermediate LNR (0.35–0.67, n = 27), and high LNR (0.68–1.0, n = 19) tiers and then sorted within each tier based on total lymph node counts, as shown in Table 4. In total, 33.8% of patients in the low LNR tier, 48.1% in the intermediate LNR tier, and 73.7% in the high LNR tier developed a recurrence. Local and distant recurrences as grouped by LNR tier are detailed in the Supplementary Material. There were no significant differences in number of lymph nodes examined between patients who did or did not develop a recurrence in any of the low LNR (*p* = 0.39), intermediate LNR (*p* = 0.47), or high LNR (*p* = 0.67) tiers.

## Discussion

The treatment of breast cancer has advanced significantly since the days of Halsted’s radical mastectomy as a primary surgical therapy.^18^ Improvements in systemic therapy for breast cancer have enabled more nuanced approaches to surgical and radiation therapy without compromising patient outcomes. Despite these advances, debate continues about the optimal management of the axilla after NAC in node-positive disease. We conducted retrospective analyses to assess the association between lymph node counts across several LNR tiers and recurrence in a cohort of young women with node-positive breast cancer who received NAC.

It is notable that 36 of 78 (46.2%) patients who experienced recurrence had HR+/HER2- disease; in other words, 36 of 113 (31.8%) patients with HR+/HER2- disease recurred within our study follow-up period. These findings may speak to the more aggressive tumor biology in the young population and to the poorer response to NAC in HR+ cancers.^11,19^ Additionally, our study spanned the years from 2010 through 2023, during which therapies such as adjuvant CDK4/6 inhibitors that improve disease-free survival would not have been routinely used until the later years of our study.^20^ Lastly, high recurrence rates in a similar population have been described in the literature. Vuong et al.^21^ examined recurrence in a population of young women with stage I–III breast cancer from 2008 to 2018. Taking all stages together, those with HR+/HER2- disease had recurrence rates of up to 20.9%. Their population was particularly diverse, including 7.8% Black, 29.4% Asian/Pacific Islander, and 19.6% Hispanic patients.^21^ In contrast, it is worth pointing out that 24.5% of our cohort was Black, a population known to experience higher recurrence rates and worse oncologic outcomes, even when controlling for tumor subtype and genomic differences.^22^

Patients who developed a recurrence presented with more advanced disease, with a significantly greater proportion of pathologic T3 disease than those who did not develop a recurrence. Similarly, although the majority of patients had clinical N1 disease in both groups, a higher proportion of patients had clinical N3 disease among those who developed a recurrence than among those who did not develop a recurrence. Patients who recurred also had a greater residual burden of disease after chemotherapy in both the breast and the axilla. A greater proportion of patients achieved ypT0 and ypN0 status after NAC among those who did not develop a recurrence than among those who did.

Rates of adjuvant radiation therapy, targeted therapy, and chemotherapy were similar between patients who did and did not develop a recurrence. There was a significantly higher rate of adjuvant endocrine therapy (*p* = 0.04) among patients who did not develop a recurrence than among those who did. Further studies are required to elucidate this effect, such as granular analyses of patient adherence.

The proportion of patients who developed a recurrence increased with higher LNR, consistent with prior reports.^16,17^ More importantly, within each LNR tier, there were no significant differences in the total count of examined lymph nodes, whether three or >16, between patients who did and did not develop a recurrence among those with a minimum of one positive lymph node of a minimum of three examined lymph nodes.

This study had several limitations. The analysis of a small cohort at a single institution limits statistical power and generalizability. Future studies would benefit from investigation of larger cohorts at multiple sites. Additionally, this was a retrospective review of data spanning a 14-year period, during which many standards of care for both systemic and radiation therapy have evolved. Prospective data are needed. Alliance for Clinical Trials in Oncology A11202 is prospectively examining the outcomes of ALND plus radiation versus ALND alone after NAC in node-positive women.^23^ The study has completed accrual; we eagerly look forward to those results.

Furthermore, the median follow-up period in this study was relatively limited at 60 months. Longer follow-up periods would improve the assessment of recurrence and survival in future studies, which is particularly important in this younger patient population. Additional subgroup analysis, such as by tumor subtype, or more granular analyses of those with isolated tumor cells or microinvasive nodal disease, as well as comparisons with older age cohorts, would provide greater mechanistic insights.

Lastly, for the subset of patients with HR+/HER2- disease, the use of genomic assays at the time of diagnosis or in the neoadjuvant setting could be further explored.^24,25^ The use of genomic assays to predict the benefit of chemotherapy is relatively recent, but evidence continues to build that oncologic outcomes reflect tumor biology more so than tumor burden.^26,27^ Patients with a low likelihood of response to chemotherapy are more commonly being offered upfront surgery if resectable. At the same time, for premenopausal, node-positive patients, it remains unclear whether any benefit derived from chemotherapy is due to the chemotherapy itself or the induced ovarian function suppression; OFSET is examining this question and continues to accrue.^28^ Both these areas of investigation would help to guide decisions on chemotherapy and personalize treatment plans for the young, node-positive, HR+/HER2- population.

To our knowledge, this is the first such study to examine lymph node involvement after NAC and recurrence at the level of positive and total lymph node counts. Although further study is needed, our findings indicate that oncologic outcomes may be more reflective of tumor biology, chemoresistance, and residual tumor burden after NAC than the number of nodes removed or the extent of nodal surgery. Prospective, large-scale, long-term studies are needed to better elucidate this question, especially in the young, high-risk population.

## Supplementary Information

Below is the link to the electronic supplementary material.

Supplementary file1 (DOCX 101 KB)
