# Supplementary material for: Impact of Total and Positive Lymph Node Count on Recurrence in Young Women with Node-Positive Breast Cancer Undergoing Neoadjuvant Chemotherapy
Source: Ann Surg Oncol. 2026 Jul 8;33(11):9888–95. doi: 10.1245/s10434-026-20154-w (PMC13630816; doi:10.1245/s10434-026-20154-w)
Supplement: Supplementary file 1 [file 10434_2026_20154_MOESM1_ESM.docx]

**SUPPLEMENTARY MATERIAL**

|  | Recurrence (n=52) | | | p value |
| --- | --- | --- | --- | --- |
|  | Total (%) | Local | Distant | 1.0 |
| Lymph node ratio (LNR) |  |  |  |  |
| LNR 0.01-0.34 |  |  |  |  |
| 3 lymph nodes examined | 2 | 0 | 2 |  |
| 4 | 3 | 1 | 2 |  |
| 5 to 9 | 6 | 1 | 5 |  |
| 10 to 15 | 9 | 1 | 8 |  |
| 16+ | 5 | 0 | 5 |  |
| Total | 25 (48.1) | 3 | 22 |  |
| LNR 0.35-0.67 |  |  |  |  |
| 3 lymph nodes examined | 0 | 0 | 0 |  |
| 4 | 1 | 0 | 1 |  |
| 5 to 9 | 4 | 1 | 3 |  |
| 10 to 15 | 7 | 0 | 7 |  |
| 16+ | 1 | 0 | 1 |  |
| Total | 13 (25.0) | 1 | 12 |  |
| LNR 0.68-1.0 |  |  |  |  |
| 3 lymph nodes examined | 2 | 0 | 2 |  |
| 4 | 2 | 0 | 2 |  |
| 5 to 9 | 4 | 0 | 4 |  |
| 10 to 15 | 2 | 0 | 2 |  |
| 16+ | 4 | 1 | 3 |  |
| Total | 14 (26.9) | 1 | 13 |  |
|  |  |  |  |  |

**Table S1. Local and distant recurrences by lymph node ratio**. Statistical analysis performed using Fisher’s exact test

**Figure S1. Overall survival (OS) by lymph node ratio (LNR)**

| **LNR** | **Median OS** | **Lower 95% Confidence Limit** | **Upper 95% Confidence Limit** |
| --- | --- | --- | --- |
| LNR 0.01-0.34 | 139.403 | 128.167 | . |
| LNR 0.35-0.67 | . | 68.601 | . |
| LNR 0.68-1.0 | 76.388 | 45.175 | . |

**Figure S2. Recurrence-free survival (RFS) by lymph node ratio (LNR)**

| **LNR** | **Median RFS** | **Lower 95% Confidence Limit** | **Upper 95% Confidence Limit** |
| --- | --- | --- | --- |
| LNR 0.01-0.34 | . | 106.516 | . |
| LNR 0.35-0.67 | 117.095 | 35.845 | . |
| LNR 0.68-1.0 | 24.953 | 18.596 | 61.734 |

**Figure S3. Progression-free survival (PFS) by lymph node ratio (LNR)**

| **LNR** | **Median PFS** | **Lower 95% Confidence Limit** | **Upper 95% Confidence Limit** |
| --- | --- | --- | --- |
| LNR 0.01-0.34 | . | 69.751 | . |
| LNR 0.35-0.67 | 81.184 | 35.845 | . |
| LNR 0.68-1.0 | 24.953 | 18.596 | 61.734 |
